# Identification of a neutralizing linear epitope within the VP1 protein of coxsackievirus A10

**DOI:** 10.1186/s12985-022-01939-3

**Published:** 2022-12-01

**Authors:** Hanyu Zhu, Xin Liu, Yue Wu, Yunyi He, Huanying Zheng, Hongbo Liu, Qiliang Liu

**Affiliations:** 1 College of Intelligent Medicine and Biotechnology, Guilin Medical University, Guilin, Guangxi China; 2 Department of Laboratory Medicine, The Second Affiliated Hospital of Guilin Medical University, Guilin, Guangxi China; 3 Guangxi Health Commission Key Laboratory of Glucose and Lipid Metabolism Disorders, Guilin, Guangxi China; 4 Guangdong Provincial Institute of Public Health, Guangdong Provincial Center for Disease Control and Prevention, Guangzhou, Guangdong China; 5 Key Laboratory of Medical Biotechnology and Translational Medicine (Guilin Medical University), Education Department of Guangxi Zhuang Autonomous Region, Guangxi, China

**Keywords:** Coxsackievirus A10, Epitope, Neutralization, Bioinformatics

## Abstract

**Background:**

Coxsackievirus A10 (CV-A10) is a leading cause of hand, foot, and mouth disease (HFMD). It is necessary to identify neutralizing epitopes to investigate and develop an epitope-based vaccine against CV-A10. The viral protein VP1 is the immunodominant capsid protein and contains the critical neutralizing epitope. However, neutralizing epitopes within VP1 protein of CV-A10 have not been well characterized.

**Methods:**

Bioinformatics techniques were applied to predict linear epitopes on the CV-A10 VP1 protein. The advanced structural features of epitopes were analyzed by three-dimensional (3D) modeling. The anticipated epitope peptides were synthesized and used to immunize mice as antigens. ELISA and micro-neutralization assay were used to determine the specific IgG antibody and neutralizing antibody titers. The protective efficacy of the epitope peptides *in viv*o was evaluated using a passive immunization/challenge assay.

**Results:**

Three linear epitopes (EP3, EP4, and EP5) were predicted on CV-A10 VP1, all spatially exposed on the capsid surface, and exhibited adequate immunogenicity. However, only EP4, corresponding to residues 162–176 of VP1, demonstrated potent neutralization against CV-A10. To determine the neutralizing capacity of EP4 further, EP4 double-peptide was synthesized and injected into mice. The mean neutralizing antibody titer of the anti-EP4 double-peptide sera was 1:50.79, which provided 40% protection against lethal infection with CV-A10 in neonatal mice. In addition, sequence and advanced structural analysis revealed that EP4 was highly conserved among representative strains of CV-A10 and localized in the EF loop region of VP1, like EV-A71 SP55 or CV-A16 PEP55.

**Conclusions:**

These data demonstrate that EP4 is a specific linear neutralizing epitope on CV-A10 VP1. Its protective efficacy can be enhanced by increasing its copy number, which will be the foundation for developing a CV-A10 epitope-based vaccine.

**Supplementary Information:**

The online version contains supplementary material available at 10.1186/s12985-022-01939-3.

## Background

Coxsackievirus A10 (CV-A10) is a human enterovirus belonging to the A species of the *Enterovirus* genus within *Picornaviridae* family [1]. CV-A10-associated hand, foot, and mouth diseases (HFMD) typically manifest mild and self-limiting symptoms. Rarely, severe clinical manifestations, such as herpangina, acute hemorrhagic conjunctivitis, acute respiratory tract infections, viral myocarditis, and even death, are observed in a few cases [2, 3]. Recently, the increasing incidence of CV-A10 infection has been an alarming trend [4]. In addition, the co-circulation of CV-A10 with other human enteroviruses may increase the likelihood of co-infection and the risk of EV genetic recombination. However, there is no specific medicine or vaccine registered against CV-A10. Several CV-A10 vaccines, including inactivated vaccines, virus-like particles (VLPs), and VLPs derived from non-EV-core viruses, have been developing for years [5].

CV-A10 is a non-enveloped icosahedral particle with a diameter of approximately 24–30 nm. The genome is approximately 7500 nt in length and consists of a single stranded positive sense RNA genome with two open reading frames (ORF1 and ORF2) [6]. ORF1 encodes a polyprotein. Further cleavage of polyprotein generates three precursor proteins. Four structural proteins (VP1, VP2, VP3, and VP4) derived from the precursor protein P1 are assembled to form the virus capsid [7]. P2 and P3 are subdivided into the nonstructural viral proteins, including 2A, 2B, 2C, 3A, 3B, 3C, and 3D [8].

Studies have shown that multiple protruding loops, such as B–C loop (residues 97–105), E–F loop (residues 163–177), G-H loop (residues 208–225), and proximity of C-terminus (residues 253–267) in VP1 of EV-A71 have been identified as the significant antigenic proteins exposed on the viral surface [9–12]. The neutralizing epitopes in these loop structures have been extensively characterized by neutralizing antisera and monoclonal antibodies. The results indicate that VP1 protein of enterovirus contains potential neutralizing epitopes and is the primary target for diagnostic reagents and vaccine research [13–15]. However, neutralizing epitopes in CV-A10 VP1 have not been experimentally confirmed.

The conventional approach for identifying immunodominant linear neutralizing epitopes on the viral capsid is made by screening a peptide library covering the entire protein sequences. These peptides are analyzed with the polyclonal or monoclonal antibody, followed by the determination of their neutralization-inhibitory effects [6, 16], which are generally considered expensive and time consuming. Bioinformatics can be utilized to rapidly predict linear epitopes and present their advanced structural characteristics [17, 18]. When combined with immunological techniques, the efficiency and precision of identifying epitopes can be improved [19]. Current linear epitope prediction includes the single parametric method and the multi-parameter synthetic prediction method utilizing online servers, such as ABCpred, BepiPred, and SVMTrip [20]. The physicochemical properties of epitopes, including secondary structure, hydrophilicity, flexibility, antigenicity, and surface accessibility, can be analyzed by PSIPRED and DNA STAR software. Furthermore, the location of epitopes can be represented visually by three-dimensional (3D) models. These techniques have been extensively applied to investigate the epitopes of influenza virus, hepatitis A virus, SARS-CoV-2, and other pathogens [21–23].

In the current study, bioinformatics techniques were applied to predict three candidate linear neutralizing epitopes within the VP1 protein of CV-A10. The candidate epitope peptides were subsequently synthesized and conjugated with keyhole limpet hemocyanin (KLH) for the immunization of mice. The levels of specific IgG and neutralizing antibodies were evaluated in the sera of mice. EP4, a highly neutralizing epitope peptide, was identified as a potential neutralizing epitope. Notably, EP4 double-peptide demonstrated enhanced neutralizing protection, which could passively protect neonatal mice from lethal CV-A10 challenges, making it a promising candidate for CV-A10 vaccine.

## Methods

### Cells and virus

Human Rhabdomyoma (RD) cells were cultured in Dulbecco modified eagle medium (DMEM) containing 10% fetal bovine serum (FBS). CV-A10 strain used in this study was the isolate P148/ZS/CHN/2012 (GenBank: MK645898.1).

### Linear epitope prediction and secondary structure analysis of CV-A10 VP1 protein

To predict the linear neutralization epitopes of CA-V10 VP1, the amino acid sequence of CV-A10-P148 VP1 protein was obtained from the GenBank database (GenBank ID: QEO24698.1). ABCPreds (http://crdd.osdd.net/raghava/abcpred/), BCPreds (http://ailab.ist.psu.edu/bcpred/) and SVMTriP (http://sysbio.unl.edu/SVMTriP/prediction.php) were used to predict the possible linear neutralizing epitopes. PSIPRED 4.0 and DNA Star Protean modules [24, 25] were utilized to analyze the secondary structure, flexibility, hydrophilicity, surface accessibility, and antigenicity of VP1 protein. According to the epitope prediction and the analysis results of physicochemical properties, a peptide meeting the following conditions was selected as a candidate epitope: (1) it should be located in the high score region predicted by the server; (2) it should avoid α-helical or β-strand structure; (3) it should show good hydrophilicity, flexibility, and antigenicity; (4) loop structures that are exposed to the outside surface are preferentially selected.

### Homology modeling and epitope spatial displaying

Homologous sequences were retrieved using the automatic mode of SWISS-MODEL, then the Cryo-EM structure of CV-A10 mature virion (PDB:6IIJ) was selected as a modeling template. The VP1, VP2, VP3, and VP4 amino acid sequences of CV-A10-P148 were submitted to a new modeling project. The PyMOL program was used to visualize the 3D model structure and display the position of the epitope.

### Synthetic peptides

All peptides were synthesized by Sener Biotechnology Co., LTD (Hefei, China). An irrelevant peptide (QLINTNGSWHINSTA) from the hepatitis C virus E2 protein was synthesized as a negative control [4]. The purity of all synthetic peptides was > 95%. For immunization, all synthetic peptides were conjugated to KLH.

### Mice immunization and serum sample collection

All animal experiments were conducted strictly with experimental protocols approved by the Animal Ethics Committee at Guilin Medical College. BALB/c mice produced in specific pathogen-free (SPF) conditions for this study were provided by SJA Laboratory Animal Co., LTD (Hunan, China). Six-week-old female BALB/C mice were randomly divided into groups with six mice per group. The experimental group, negative control group, and blank group mice were multi-point subcutaneously injected with synthetic peptides (100 µg/dose), irrelevant peptides (100 µg/dose), or PBS, respectively. The first injection of each sample was emulsified with an equal volume of complete Freund's adjuvant (Sigma), and the booster was emulsified with an equal volume of incomplete Freund's adjuvant. Serum samples were harvested two weeks after the last immunization [16].

### Peptide-ELISA

The peptide-specific IgG antibody in immunized mouse sera was determined by indirect ELISA assay. Briefly, each well in a 96-well plate was coated with 10 ng of a synthetic epitope at 4 °C overnight and then was blocked with 1% bovine serum albumin (BSA) for 1 h at 37 °C. The plates were incubated at 37 °C for 1 h with epitope peptide antisera that diluted by two-fold starting at 1:500 in PBS-Tween-20 (PBST) containing 0.1% BSA and then was incubated with HRP-conjugated goat anti-mouse IgG antibody that diluted at 1:5000 in PBST at 37 °C for 1 h. The plates were washed three times with PBST between each step. After color development, the optical density (OD) was measured on a microplate reader (Bio-Rad) at 450 nm.

### Micro-neutralization assay

Referring to previous research [26], the titer of CV-A10 neutralizing antibody in the sera was determined using the cytopathic effect (CPE)-based micro-neutralization assay on RD cells. The neutralization antibody titer was defined as the reciprocal of the highest serum dilutions that could protect 50% of cells from CPE.

### In vivo protection analysis

Enterovirus-infected neonatal mouse model is a classic model commonly used in the evaluation of vaccines or antiviral drugs [27, 28]. A standardized CV-A10- infected neonatal mouse model [29] was used to evaluate the protective effects of EP4 double-peptide against a challenge with the mouse adapted CV-A10 strain. Since the immune system of newborn mice is not yet mature enough to elicit immune responses against foreign antigens, protective efficacy was determined by passive immunization/challenge assay [30]. Adult ICR mice were grouped and injected using the same method and dose identical to that used in the immunogenicity experiment. Mice were mated one week after the first booster, and each mouse gave birth to about 10 suckling mice about three weeks after mating. Neonatal mice were challenged intracranially with 50 × LD50 of CV-A10 within 24 h after delivery. They were injected intraperitoneally with pooled anti-EP4 double-peptide sera, anti-irrelevant peptide sera, or anti-PBS sera within 2 h after the challenge. The clinical symptoms and death of mice in each group were monitored for 15 consecutive days.

### Sequence alignment and advanced structure analysis

All VP1 protein sequences of different enteroviruses for sequence alignment were retrieved from GenBank. The alignment of VP1 protein sequences was conducted using BioEdit software. The PyMOL program was used for the superposition of VP1 3D models of CV-A10 (PDB: 6IIJ), EV-A71 (PDB: 4aed), and CV-A16 (PDB: 5c4w). The linear neutralizing epitopes were tagged on 3D models of each VP1, respectively.

### Statistical analysis

GraphPad Prism 8 software was used for graph generation and statistical analyses. Log-rank test was employed to compare Kaplan Meier survival curves, and the student's two-tailed *t*-test was utilized to analyze serum-specific IgG antibody titer and neutralizing antibody titer. A *p*-value of less than 0.05 was considered statistically significant.

## Results

### Prediction results of the B cell linear epitopes of CV-A10 VP1

B cell linear epitopes are crucial in eliciting humoral immune protection [15]. The virus cannot invade cells if neutralizing antibodies induced by linear neutralizing epitopes are present. Several servers based on neural algorithms and polypeptide feature analysis were implemented to improve the accuracy of epitope prediction. Five candidate linear epitopes on CV-A10 VP1 (Table 1) were selected for further investigation based on the results of ABCpred, BCPred, and SVMTrip servers (Additional file 1: Table S1) and analysis of their secondary structures, flexibility, antigenicity, and hydrophilicity (Additional file 1: Fig. S1).

**Table 1:** Five possible linear epitopes on CV-A10 VP1 protein

Peptides	Sequence location	Amino acid sequence
EP1	Residue 21–36	SSATNVESAANTTPSS
EP2	Residue 50–63	AETGATSNATDENM
EP3	Residue 100–107	TDTTGYAT
EP4	Residue 162–176	PTGRDAFQWQTATNP
EP5	Residue 207–224	YPTFGQHPETSNTTYGLC

### The localization of candidate linear epitopes on viral capsid

**Fig. 1:**
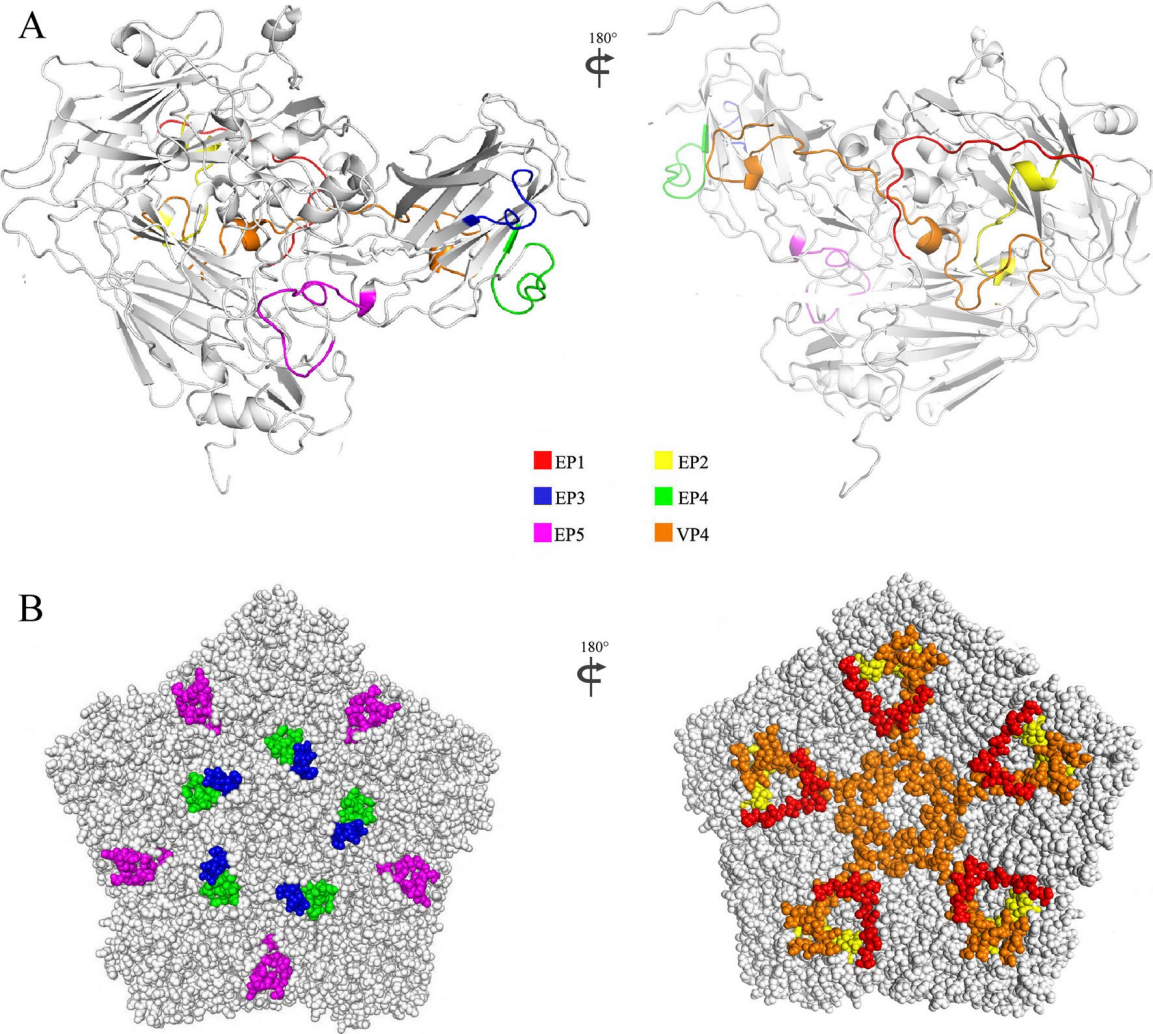
Location of predicted linear epitopes on CV-A10 P1 protein. EP1-EP5 peptide and VP4 protein are colored in red, yellow, blue, green, purple, and orange, respectively. **A** The tertiary structure is constructed with CV-A10 P1 amino acid sequence. **B** The top (left) and the bottom (right) of CV-A10 P1 pentamer are shown as a space fill model, where EP3, EP4, and EP5 are exposed on the outer capsid surface, and EP1, EP2, and VP4 proteins are in the inner surface capsid

The pentamer spatial model of CV-A10 capsid was developed using SWISS-MODEL (Fig. 1). VP4 and five candidate linear epitopes of VP1 were labeled on the model. VP4 protein is located on the inner surface of the capsid. EP1 and EP2 are also located on the inner surface, whereas EP3, EP4, and EP5 are on the outer surface. Neutralizing epitopes, which mediate virus-receptor interactions, were typically located on the external surface of the viral capsid near the vertices; therefore, EP3, EP4, and EP5 were favored as candidate linear epitopes of CV-A10 VP1.

### Analysis of mice IgG responses elicited by synthetic epitopes

**Fig. 2:**
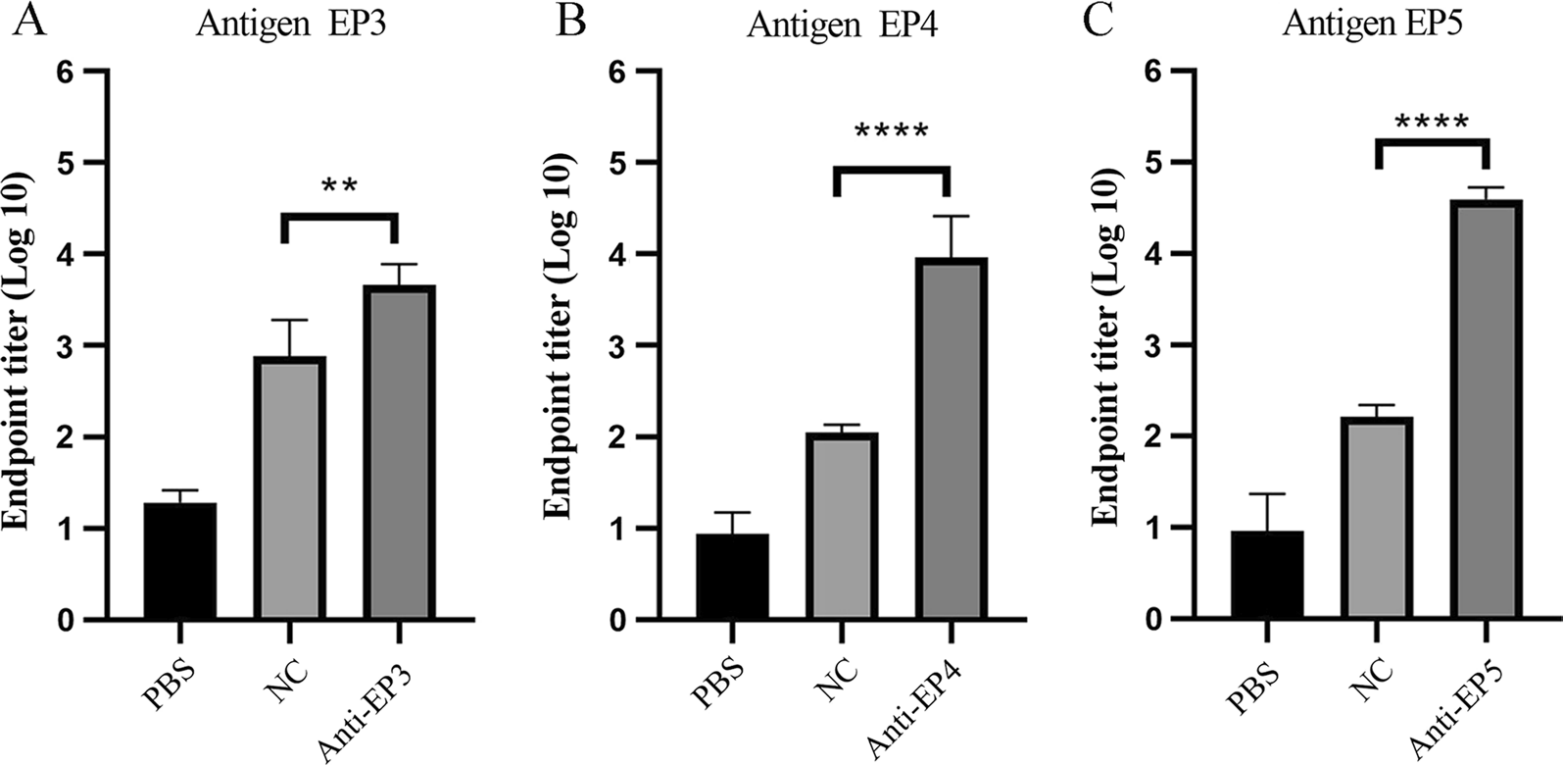
Peptide-specific IgG antibody levels in the sera obtained from immunized mice. Five adult female BALB/c mice received priming injection at week 0 and boosting injection at weeks 2 and 4 with PBS, irrelevant peptide, EP3, EP4, or EP5, respectively. The end-point dilution titers were tested by peptide ELISA using EP3 (**A**), EP4 (**B**), or EP5 (**C**) peptide as the corresponding antigen. Anti-PBS sera served as blank control, and anti-irrelevant peptide sera served as the negative control (NC). The data are presented as mean ± standard deviation (SD) of at least triplicate wells, and the means of IgG titers were compared using a student's two-tailed *t*-test. **p < 0.01, ****p < 0.0001

For the detection of immunogenicity of the linear epitopes, the three peptides (EP3, EP4, and EP5) were chemically synthesized and conjugated to KLH and administered to mice for immunization. IgG antibody levels in the sera obtained from immunized mice were determined using ELISA with a corresponding synthetic peptide as the capture antigen. The sera of mice immunized with PBS served as blank, whereas the sera of mice immunized with irrelevant peptides served as the negative control. The negative control or blank antisera exhibited no significant reactivity with any peptides. As illustrated in Fig. 2, all antiserum samples against EP3, EP4, or EP5 contained significantly higher IgG levels than the negative control. EP5 demonstrated the highest immunogenicity. The differences in OD values between groups suggest that EP3, EP4, and EP5 may induce varying degrees of immune response in mice.

### In vitro neutralizing capacity of the candidate linear epitopes antisera

**Fig. 3:**
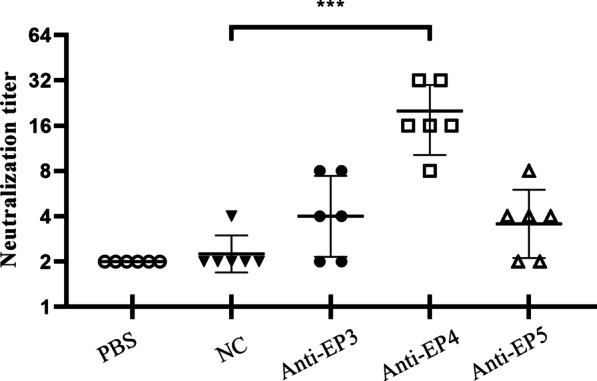
CV-A10-neutralizing antibody titration in anti-peptide sera from immunized mice. Two weeks after the final immunization, anti-peptide sera from individual mice were collected and tested for CV-A10 virus neutralization. Each symbol represents a serum sample, and the horizontal line indicates geometric mean values. According to the statistical analysis, there was a significant difference in neutralizing antibody titers between anti-EP4 sera and negative control sera. The data were analyzed using a student's two-tailed *t*-test. ***p < 0.001

**Fig. 4:**
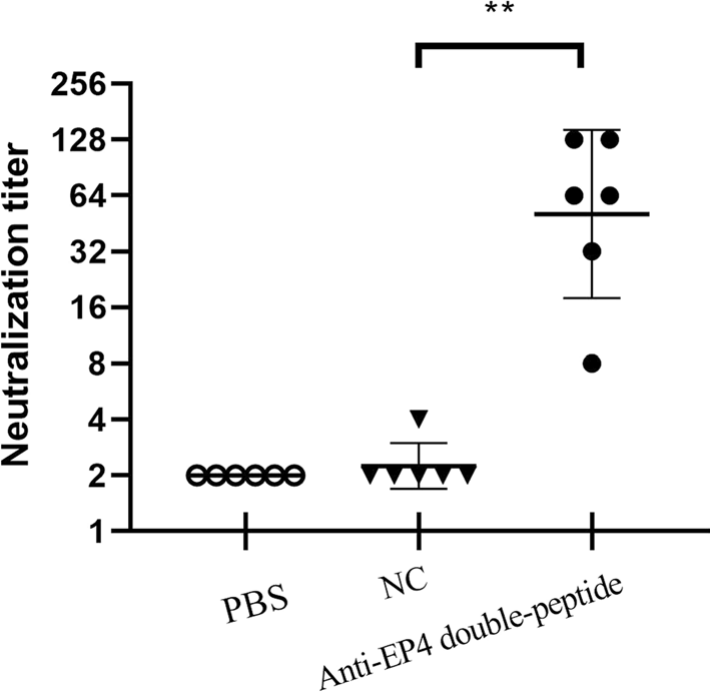
The neutralization titer of anti-EP4 double-peptide sera. Mice were immunized with EP4 double-peptide and given three booster injections two weeks apart. Sera were obtained two weeks after the final immunization for micro-neutralization experiment. The geometric mean neutralizing antibody titer of anti-EP4 double-peptide sera against CV-A10 reached 1:50.79, and a significant difference was shown between anti-EP4 double-peptide sera and negative control sera. The data were analyzed using a student's two-tailed *t*-test. **p < 0.01

The micro-neutralization assay was utilized to assess the ability of the candidate linear epitope antisera to neutralize CV-A10 infection. As demonstrated in Fig. 3, EP3, EP4, and EP5 antisera neutralized CV-A10 P148 strain with geometric mean titers of 1:4, 1:17.96, and 1:3.56, respectively. In contrast, the control antisera lacked CV-A10 neutralizing activity. There were statistically significant differences between the neutralizing capacity of groups immunized with EP4 and negative control. Since the neutralizing antibody titer elicited by the single epitope peptide was low, mice were immunized with a double-peptide EP4 to determine if EP4 is a dominant neutralizing epitope. Two weeks after three booster injections, sera for the micro-neutralization experiment were collected. As depicted in Fig. 4, sera from EP4 double-peptide group neutralized CV-A10 with titers ranging from 8 to 128, as measured by dilution.

### The in vivo protective efficacy of anti-EP4 double-peptide sera against lethal CV-A10

**Fig. 5:**
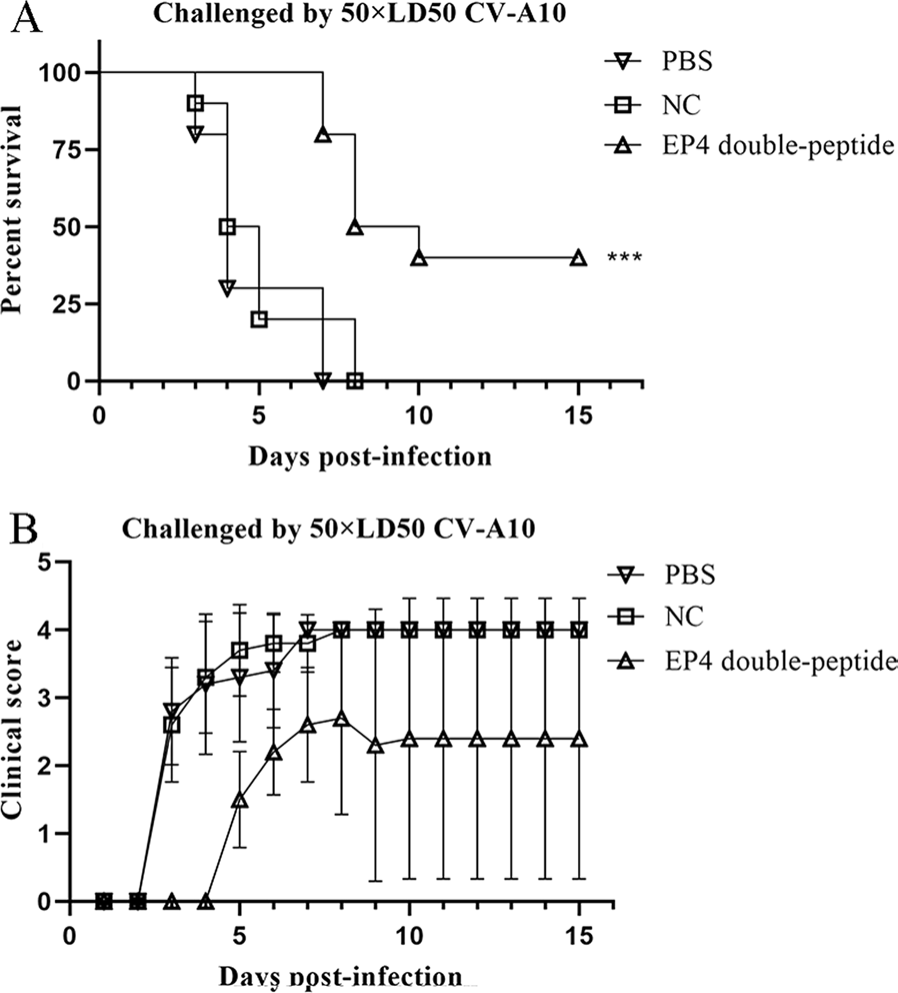
In vivo protective efficacy of anti-EP4 double-peptide sera against lethal CV-A10 challenge. The neonatal mice were challenged with 50 × LD50 of CV-A10 virus within 24 h after birth (each group = 10). They were then injected with anti-EP4 double-peptide sera, anti-irrelevant peptide sera, or anti-PBS sera. **A** Survival curve of the neonatal mice. **B** Clinical scores of the neonatal mice. The severity of clinical symptoms, from mild to severe, was scored in five grades: 0, healthy; 1, reduced mobility; 2, limb weakness; 3, paralysis; 4, death. The Mantel-Cox log-rank test evaluated the survival rates. The clinical scores were compared using Dunn's multiple-comparison test. ***p < 0.001

The protective efficacy of EP4 double-peptide in vivo was evaluated by a lethal dose of a mouse-adapted CV-A10 strain intracranially to ICR neonatal mice [31]. The results demonstrated that the mice treated with anti-PBS sera or anti-irrelevant peptide sera exhibited multiple symptoms, including limb-shake weakness and hind-limb paralysis, and began to die on day 3, with all mice dying by days 7 or 8, respectively (Fig. 5A). The mice treated with anti-EP4 double-peptide sera developed mild or severe clinical symptoms on day 4, and some mice died between day 7 and 10, while the rest gradually regained their health, with a 40% final survival rate on day 15 (Fig. 5A, B).

### The sequence and advanced structure analysis of EP4

**Fig. 6:**
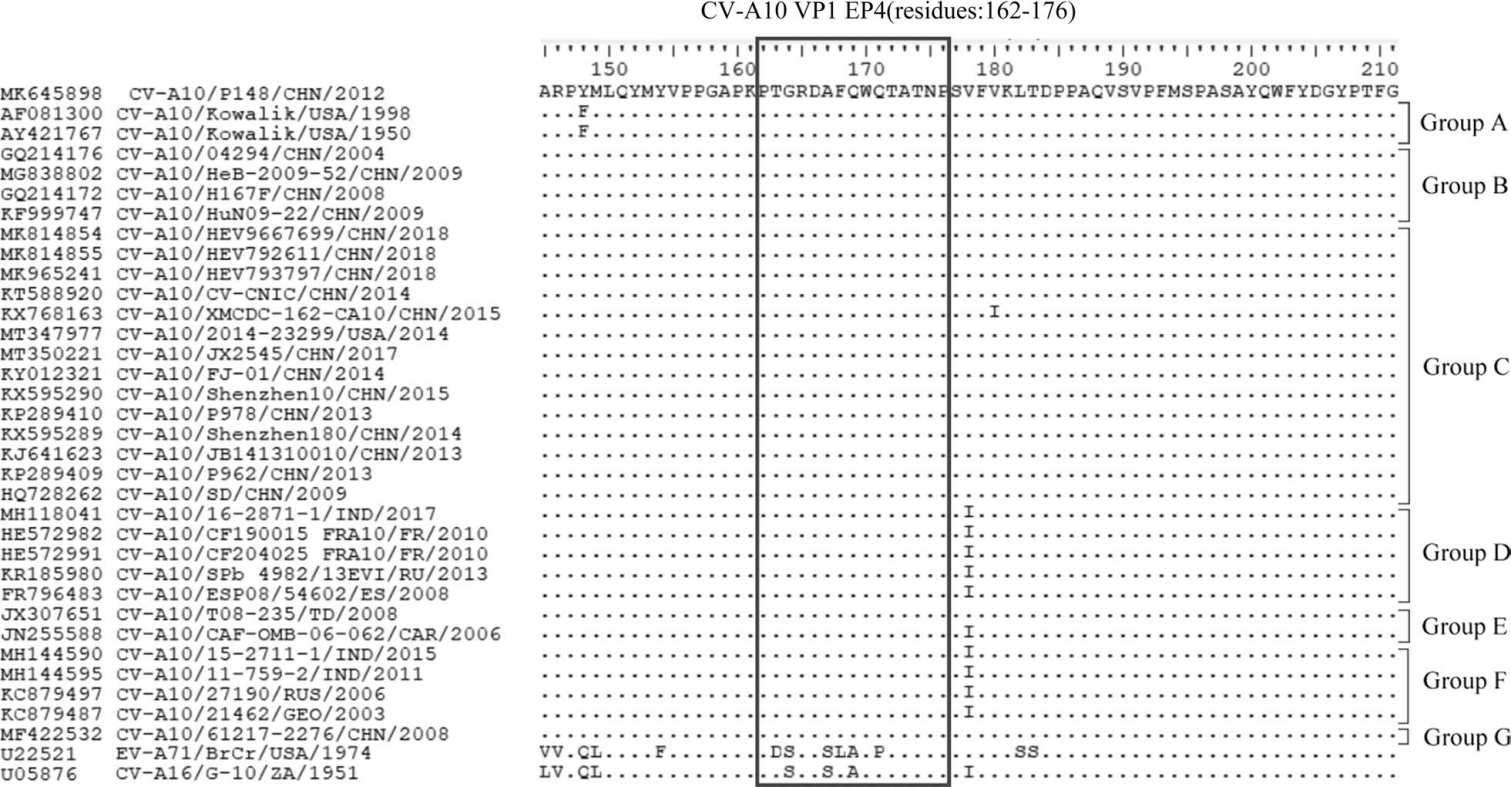
Alignment of CV-A10 VP1 amino acid sequences and the location of CV-A10 EP4. According to the evolutionary analysis of CV-A10 (Additional file 1: Fig. S2), 33 amino acid sequences of CV-A10 VP1 in group A-G were selected and compared with VP1 sequences of EV-A71 and CV-A16 prototype strains. Dots represent residues identical to CV-A10/P148/CHN/2012. EP4 region is boxed with a black line

**Fig. 7:**
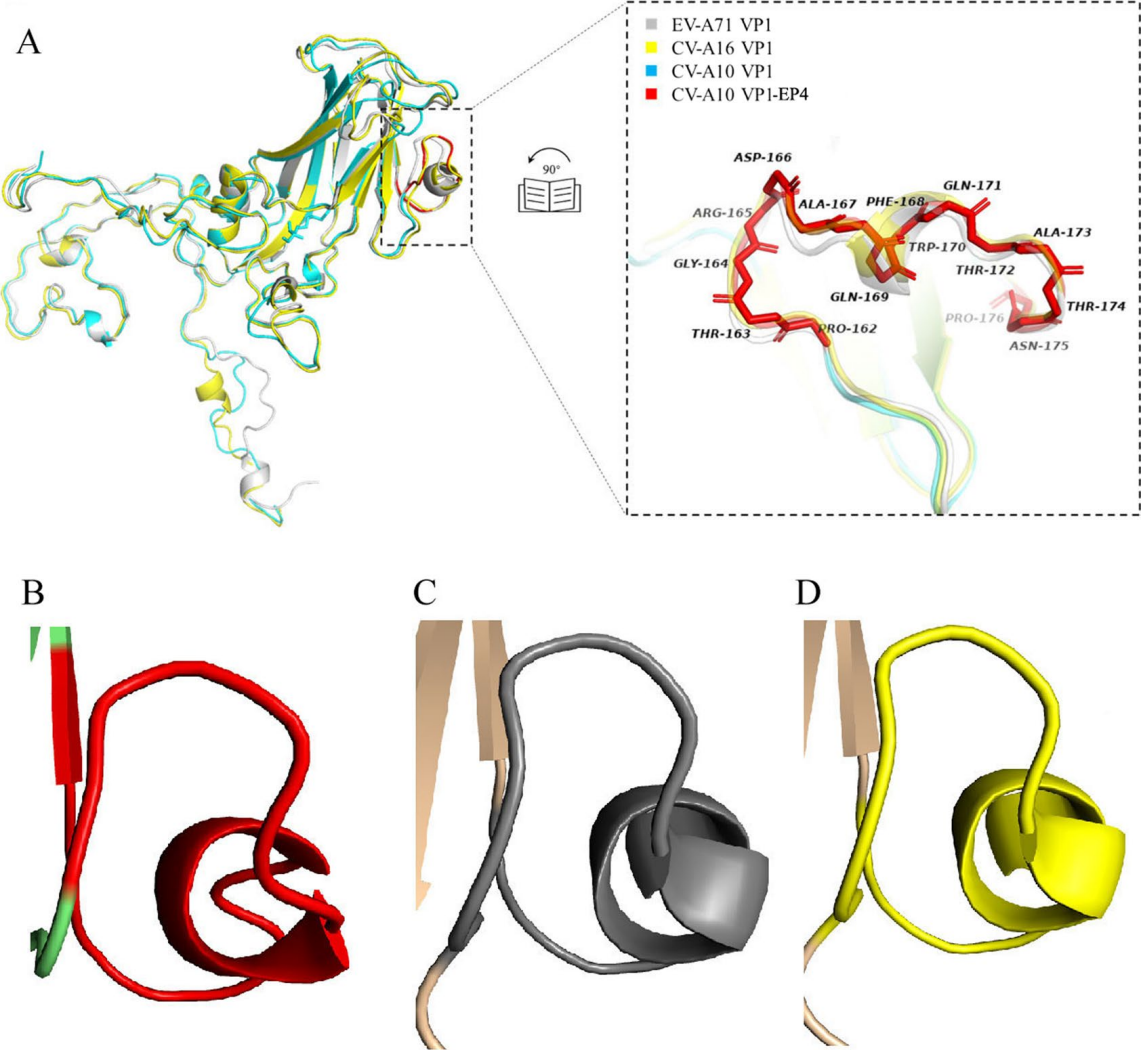
Advanced structural analysis of CV-A10 EP4, EV-A71 SP55, and CV-A16 PEP55. **A** Superposition of VP1 of CV-A10 (blue), CV-A16 (yellow) and EV-A71 (light gray). The CV-A10 EP4 is magnified and colored in red. **B**–**D** represent the secondary structure of CV-A10 EP4, EV-A71 SP55 and CV-A16 PEP, respectively

The CV-A10 VP1 sequences were analyzed by SDT software, and the phylogenetic tree was constructed using MEGA 7.0 software (Additional file 1: Fig. S2). Clustal X2 software was utilized to align the VP1 amino acid sequences of 33 representative strains of CV-A10 (A-F group) with the prototype strains of EV-A71 and CV-A16. The results indicated that EP4 region (residues 162–176 of VP1) was highly conserved among selected CV-A10 isolates. Nevertheless, EP4 was found to be 60% consistent with SP55 and 80% consistent with PEP55 (Fig. 6). EP4, SP55, and PEP55 were all located in EF loop region of VP1 and were highly exposed at the virion surface (Fig. 7A) as demonstrated by the advanced structural analysis by SWISS-MODEL. These three epitopes shared a short peptide segment (WQTATNP) that was identical. EP4 differs from SP55 and PEP55 because it has one additional random curl (Fig. 7B–D). EP4 appears to be a surface-exposed, highly conserved, neutralizing epitope on CV-A10 VP1 protein.

## Discussion

Neutralizing antibodies are elicited by the neutralizing epitopes. The level of neutralization titer is related to the amount of effective viral antigen, which is suggested as a criterion for evaluating the efficacy of vaccine candidates. Therefore, neutralizing epitopes play a key role in vaccine effectiveness and can serve as biomarkers to monitor vaccine efficacy [32]. The discovery and identification of neutralizing epitopes is critical to vaccine development.

Recently, CV-A10 has become one of the most prevalent pathogens of HFMD [33]. The linear neutralizing epitopes are essential for vaccine development of CV-A10 and its diagnosis [34]. Historically, epitopes were identified and screened by chemo-synthesizing short overlapping peptides based on their sequences. Immunological testing was then used to confirm the antigenic and immunogenic properties of each peptide. This method requires a great deal of time, human resources, and materials, and it may miss some potential neutralizing epitopes despite effectively covering the entire polypeptide chain.

In this study, online servers predicted thirteen epitopes with high scores (Additional file 1: Table S1). Based on the secondary structure analysis of VP1 protein, peptides 15, 11 and 3 with the α-helical or β-strand structure were eliminated (Additional file 1: Fig. S1A). Then, based on hydrophilicity, elasticity, antigenicity, and advanced structure of VP1, five linear neutralizing epitope candidates for CV-A10 VP1 were selected. A 3D model displayed the spatial locations of these five epitopes and demonstrated that EP1 and EP2 were embedded within the capsid, whereas EP3–EP5 were located on the exterior of the capsid. Since neutralizing epitopes are immunoreactive regions on antigens and are responsible for binding to immune cell receptors or free antibodies, they are typically found on the surface of the viral capsid. EP1 and EP2, therefore, do not meet the requirement for neutralization.

Furthermore, Dai's study demonstrated that peptides in the EP1 region are antigenic but lack neutralizing properties [6]. In addition, it is demonstrated that bioinformatics is superior to overlapping peptide mapping for locating the region that corresponds to neutralizing epitope characteristics. EP3–EP5 was synthesized and then administered to mice. ELISA demonstrated that all three candidate epitopes could induce elevated levels of self-specific IgG. EP5 induced the highest IgG titer, possibly because its spatial configuration is more conducive to the recognition by immune cells. In a micro-neutralization assay, EP4 was found to have a higher neutralizing antibody titer than EP3 and EP5. It suggests that EP4 may be a potential neutralizing epitope of CV-A10 VP1. To obtain a high-titer neutralizing antibody against EP4 and demonstrate its protective efficacy in vivo, tandem arrays of EP4 double-peptides conjugated with KLH were synthesized. The geometric mean neutralizing antibody titer against CV-A10 in antiserum from mice immunized with double-peptide EP4 was 1:50.79. To verify the passive protection efficiency of double-peptide EP4 i*n vivo*, an experimental animal model of CV-A10 infection was used [29]. The double-peptide EP4 provided 40% protection against the 50 × LD50 CV-A10 challenge in neonatal mice, whereas the PBS or the irrelevant peptide conferred no protection. After further refinement, EP4 double-peptide could be used to develop a vaccine against CV-A10 epitope based on the above results.

Studies demonstrated that EV-A71 and CV-A16 utilize human scavenger receptor class B, member 2 (hSCARB2), or human P selectin glycoprotein ligand 1 (PSGL-1) as cellular receptors [35]. The locations of epitopes in CV-A6 VP1 are comparable to those in EV-A71, including the BC loop, EF loop, GH loop, and C-terminus [9–12]. PSGL-1 was considered the cell receptor of CV-A10 [11], so the neutralizing epitopes of CV-A10 VP1 may be located in the same region as EV-A71. Sequence analysis and 3D modeling supported the hypothesis, as mentioned earlier, EP4 was located in EF loop of CV-A10 VP1 [36], the exact location as SP55 of EV-A71 VP1 and PEP55 of CV-A16 VP1. EP4 amino acids were highly conserved in CV-A10 genotype, sharing approximately 60% and 80% sequence identity with EV-A71 SP55 and CV-A16 PEP55, respectively, indicating that EP4 can also be used as a specific target for detecting CV-A10. EP4 contains three amino acids (G164S, A167S, and Q169A) different from SP55 and PEP55 that distinguish it from EV-A71 and CV-A16, suggesting that these three amino acids determine the specificity of EP4. Using a mutagenesis strategy in EP4, the relationship between EP4 and virulence, transmissibility, or pathogenicity of CV-A10 can be further investigated.

In this study, a neutralizing epitope of CV-A10 VP1 was identified. Additional bioinformatic and immunology research is required to determine if neutralizing epitopes exist in BC loop of VP1, GH loop of VP2, and GH loop of VP3. Interestingly, the immune protective range can be expanded by replacing the original epitope with a heterologous neutralizing epitope on the capsid of an EV-A71 VLP vaccine or inactivated vaccine [36, 37], suggesting that EP4 could be used as a candidate epitope for developing the novel polyvalent chimeric vaccine.

## Conclusion

On CV-A10 VP1, a highly conserved linear neutralizing epitope (EP4) has been identified. Neutralizing antibodies elicited by EP4 could protect mice from a lethal CV-A10 challenge. It can be used as a guide for designing CV-A10 epitope peptides or enterovirus polyvalent vaccines.

## Supplementary Information

**Additional file 1: Table S1** The possible linear epitopes given high scores predicted by three servers. **Figure S1**: The secondary structure analysis of CV-A10 VP1 protein. (A) The secondary structure prediction of CV-A10 VP1 protein by PSIPRED. α-Helical residues are in pink, β-strand residues are in yellow, putative domain boundaries are indicated in blue, and the locations of the predicted epitopes listed in Table S1 are underlined and numbered. (B) Graphic analysis of secondary structure, flexibility, hydrophilicity, surface accessibility and antigenicity of the CV-A10 VP1 protein using the DNAstar Protean module. **Figure S2**: Evolutionary analysis of CV-A10 VP1 protein. All available CV-A10 sequences were downloaded from GenBank and saved in “FASTA” format. Sequences with high similarity and no specific separation time and location were excluded, and representative sequences were selected for further analysis. ClustalX2 software was utilized to calibrate and compare the CV-A10 representative sequences. According to the VP1 sequence of CV-A10 prototype strain (Kowalik), the VP1 sequence of each isolate was truncated. The CV-A10 VP1 sequences were analyzed with muscle method by SDT software. The strains with nucleotide sequence consistency greater than 75% in the heat map were classified as the same genotype (A). The phylogenetic tree was inferred with the neighbor-joining (N-J) method and Kimura 2-parameter model by MEGA 7.0 software (B). The EV-A71 and CV-A16 prototype strains were used as outgroups. The reliability of the phylogenetic tree was tested by Bootstrap replication (1000) method. The tree is drawn to scale, with branch lengths in the same units as those of the evolutionary distances used to infer the phylogenetic tree. The evolutionary distances were computed using the Maximum Composite Likelihood method and are in the units of the number of base substitutions per site.

## Data Availability

Not applicable.

## Abbreviations

CV-A10: Coxsackievirus A10
CV-A16: Coxsackievirus A16
EV-A 71: Enterovirus A71
HFMD: Hand, foot and mouth disease
3D: Three-dimensional
VLPs: Virus-like particles
ORF: Open reading frame
KLH: Keyhole limpet hemocyanin
RD: Rhabdomyosarcoma
DMEM: Dulbecco’s modified eagle medium
FBS: Fetal bovine serum
SPF: Specific pathogen-free
BSA: Bovine serum albumin
PBST: PBS-Tween-20
OD: Optical density
CPE: Cytopathic effect
hSCARB2: Human scavenger receptor class B, member 2
PSGL-1: P selectin glycoprotein ligand 1
ICR: Institute of Cancer Research
TCID50: 50% Tissue culture infectious dose
