# Supplementary material for: Identification of a neutralizing linear epitope within the VP1 protein of coxsackievirus A10
Source: Virol J. 2022 Dec 1;19:203. doi: 10.1186/s12985-022-01939-3 (PMC9714398; doi:10.1186/s12985-022-01939-3)
Supplement: Supplementary file 1 — Additional file 1: Table S1 The possible linear epitopes given high scores predicted by three servers. Figure S1: The secondary structure analysis of CV-A10 VP1 protein. (A) The secondary structure prediction of CV-A10 VP1 protein by PSIPRED. α-Helical residues are in pink, β-strand residues are in yellow, putative domain boundaries are indicated in blue, and the locations of the predicted epitopes listed in Table S1 are underlined and numbered. (B) Graphic analysis of secondary structure, flexibility, hydrophilicity, surface accessibility and antigenicity of the CV-A10 VP1 protein using the DNAstar Protean module. Figure S2: Evolutionary analysis of CV-A10 VP1 protein. All available CV-A10 sequences were downloaded from GenBank and saved in “FASTA” format. Sequences with high similarity and no specific separation time and location were excluded, and representative sequences were selected for further analysis. ClustalX2 software was utilized to calibrate and compare the CV-A10 representative sequences. According to the VP1 sequence of CV-A10 prototype strain (Kowalik), the VP1 sequence of each isolate was truncated. The CV-A10 VP1 sequences were analyzed with muscle method by SDT software. The strains with nucleotide sequence consistency greater than 75% in the heat map were classified as the same genotype (A). The phylogenetic tree was inferred with the neighbor-joining (N-J) method and Kimura 2-parameter model by MEGA 7.0 software (B). The EV-A71 and CV-A16 prototype strains were used as outgroups. The reliability of the phylogenetic tree was tested by Bootstrap replication (1000) method. The tree is drawn to scale, with branch lengths in the same units as those of the evolutionary distances used to infer the phylogenetic tree. The evolutionary distances were computed using the Maximum Composite Likelihood method and are in the units of the number of base substitutions per site. [file 12985_2022_1939_MOESM1_ESM.docx]

Table of content

Page 2 ………………………………………………………………………………….. sTable 1

Page 3 ………………………………………………………………………………….. sFig 1

Page 4 ………………………………………………………………………………….. sFig 2

**sTable 1 The possible linear epitopes given high scores predicted by three servers**

| Servers | Number | Starting position | Amino acid sequence | score |
| --- | --- | --- | --- | --- |
| ABCPred | 1 | 199 | AYQWFYDGYPTFGQHP | 0.92 |
|  | 2 | 148 | YMLQYMYVPPGAPKPT | 0.92 |
|  | 3 | 170 | WQTATNPSVFVKLTDP | 0.89 |
|  | 4 | 156 | PPGAPKPTGRDAFQWQ | 0.89 |
|  | 5 | 119 | RRKCEMFTYMRFNAEF | 0.88 |
| BCPred | 6 | 153 | MYVPPGAPKPTGRDAF | 1 |
|  | 7 | 97 | DGGTDTTGYATWDIDI | 0.999 |
|  | 8 | 21 | SSATNVESAANTTPSS | 0.997 |
|  | 9 | 183 | TDPPAQVSVPFMSPAS | 0.996 |
|  | 10 | 212 | QHPETSNTTYGLCPNN | 0.99 |
|  | 11 | 233 | AVRVVSREASQLKLQT | 0.97 |
|  | 12 | 48 | QAAETGATSNATDENM | 0.959 |
| SVMTrip | 13 | 7 | IIHDALGNTVRRAISS | 1 |


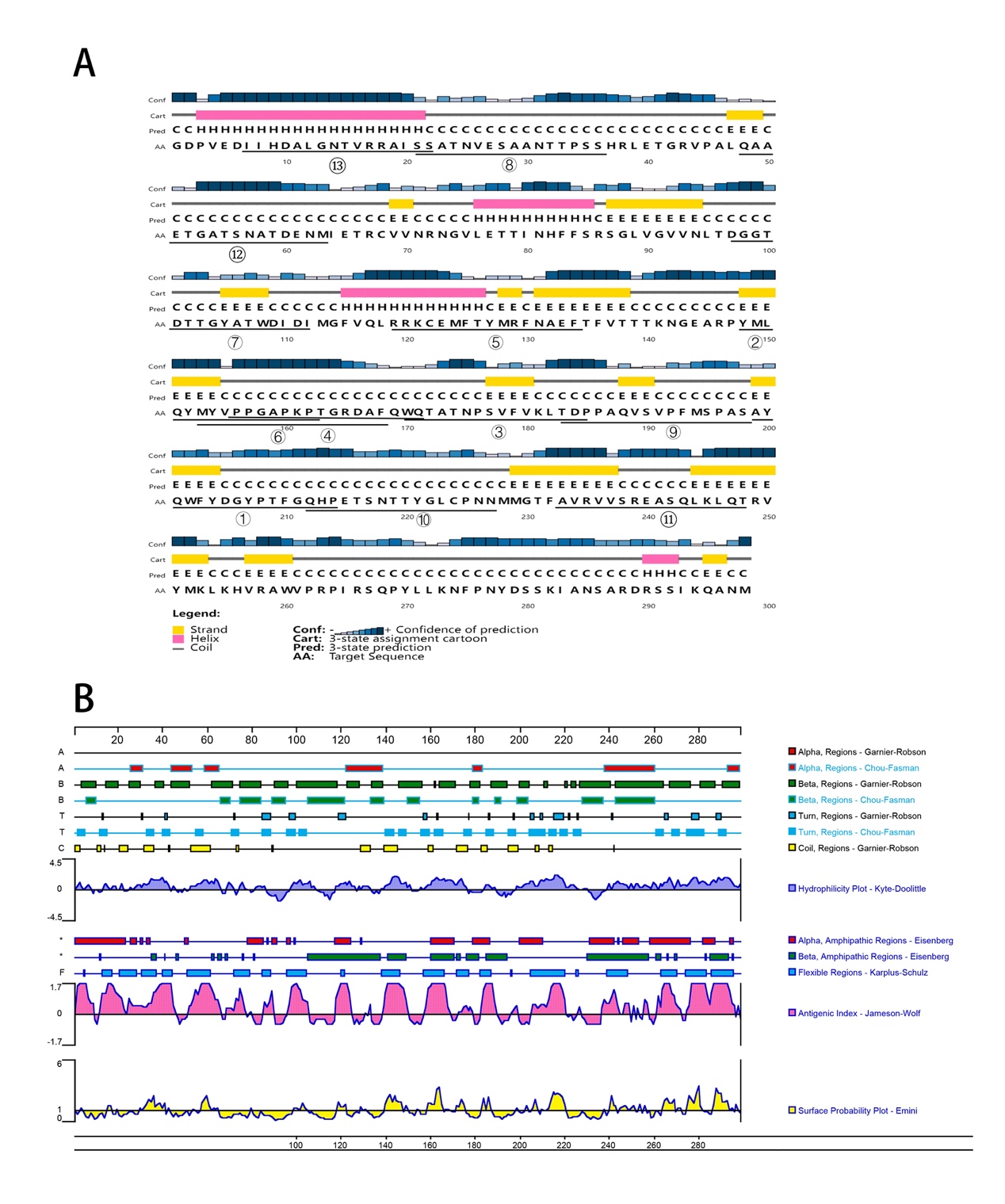


**sFig 1: The secondary structure analysis of CV-A10 VP1 protein.** (A) The secondary structure prediction of CV-A10 VP1 protein by PSIPRED. α-Helical residues are in pink, β-strand residues are in yellow, putative domain boundaries are indicated in blue, and the locations of the predicted epitopes listed in sTable 1 are underlined and numbered. (B) Graphic analysis of secondary structure, flexibility, hydrophilicity, surface accessibility and antigenicity of the CV-A10 VP1 protein using the DNAstar Protean module.


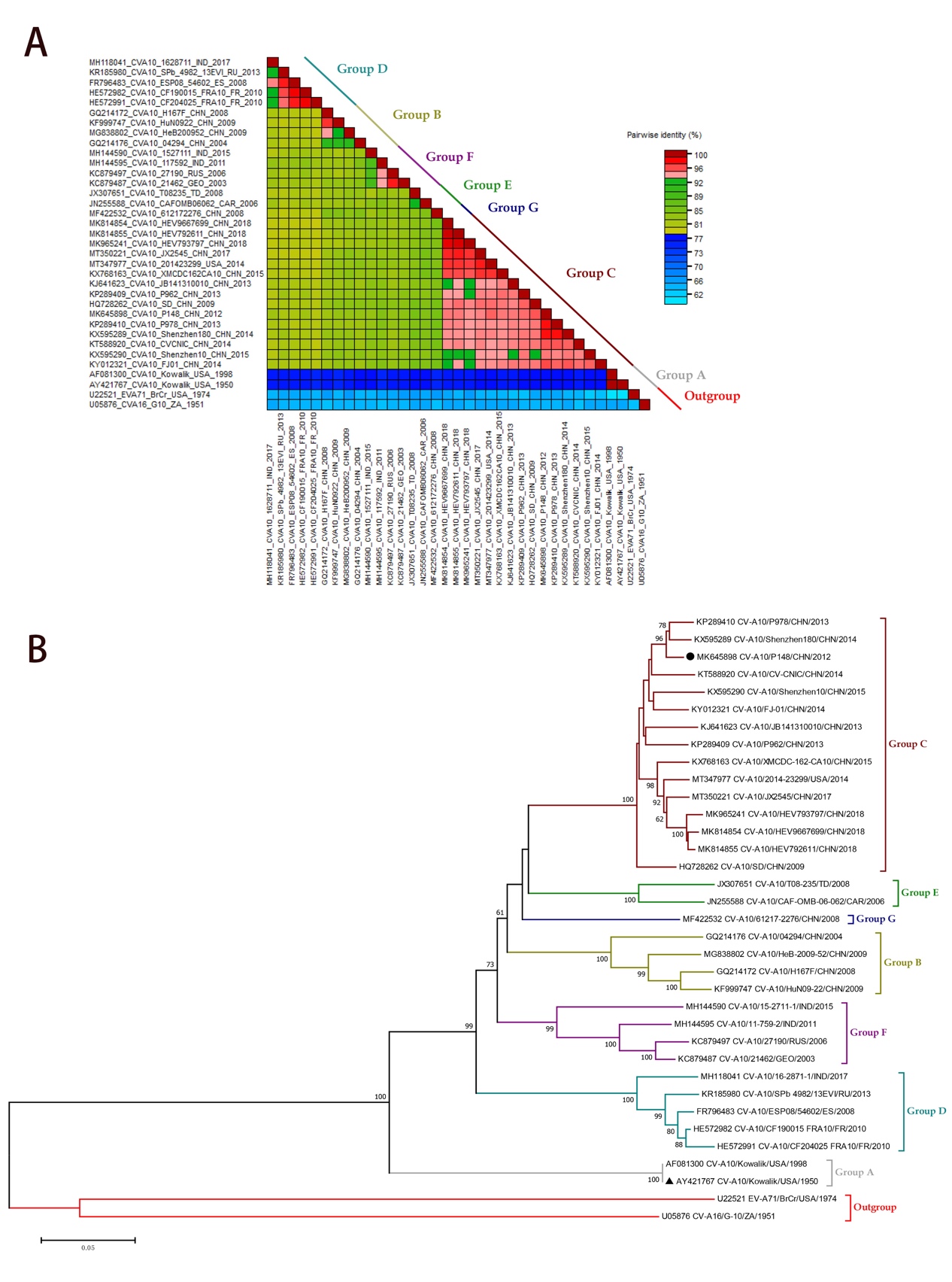


**sFig 2: Evolutionary analysis of CV-A10 VP1 protein.** All available CV-A10 sequences were downloaded from GenBank and saved in "FASTA" format. Sequences with high similarity and no specific separation time and location were excluded, and representative sequences were selected for further analysis. ClustalX2 software was utilized to calibrate and compare the CV-A10 representative sequences. According to the VP1 sequence of CV-A10 prototype strain (Kowalik), the VP1 sequence of each isolate was truncated. The CV-A10 VP1 sequences were analyzed with muscle method by SDT software. The strains with nucleotide sequence consistency greater than 75% in the heat map were classified as the same genotype (A). The phylogenetic tree was inferred with the neighbor-joining (N-J) method and Kimura 2-parameter model by MEGA 7.0 software (B). The EV-A71 and CV-A16 prototype strains were used as outgroups. The reliability of the phylogenetic tree was tested by Bootstrap replication (1000) method. The tree is drawn to scale, with branch lengths in the same units as those of the evolutionary distances used to infer the phylogenetic tree. The evolutionary distances were computed using the Maximum Composite Likelihood method and are in the units of the number of base substitutions per site.
